# Identifying signatures of sexual selection using genomewide selection components analysis

**DOI:** 10.1002/ece3.1546

**Published:** 2015-06-19

**Authors:** Sarah P Flanagan, Adam G Jones

**Affiliations:** Biology Department, Texas A&M University3258 TAMU, College Station, Texas, 77843

**Keywords:** Adaptation, population genetics, population genomics, RAD-seq, reproductive success, simulation model

## Abstract

Sexual selection must affect the genome for it to have an evolutionary impact, yet signatures of selection remain elusive. Here we use an individual-based model to investigate the utility of genome-wide selection components analysis, which compares allele frequencies of individuals at different life history stages within a single population to detect selection without requiring a priori knowledge of traits under selection. We modeled a diploid, sexually reproducing population and introduced strong mate choice on a quantitative trait to simulate sexual selection. Genome-wide allele frequencies in adults and offspring were compared using weighted *F*_ST_ values. The average number of outlier peaks (i.e., those with significantly large *F*_ST_ values) with a quantitative trait locus in close proximity (“real” peaks) represented correct diagnoses of loci under selection, whereas peaks above the *F*_ST_ significance threshold without a quantitative trait locus reflected spurious peaks. We found that, even with moderate sample sizes, signatures of strong sexual selection were detectable, but larger sample sizes improved detection rates. The model was better able to detect selection with more neutral markers, and when quantitative trait loci and neutral markers were distributed across multiple chromosomes. Although environmental variation decreased detection rates, the identification of real peaks nevertheless remained feasible. We also found that detection rates can be improved by sampling multiple populations experiencing similar selection regimes. In short, genome-wide selection components analysis is a challenging but feasible approach for the identification of regions of the genome under selection.

## Introduction

One of the most important questions in evolutionary biology is how selection, which by definition acts on phenotypes, causes heritable changes (Nielsen 2005). Recent advances in DNA sequencing technologies have provided many new opportunities to explore how genomes are affected by selection, but no method currently exists to detect the signature of individual episodes of selection within the time frame of a single generation on a genome-wide scale. Yet, we know that total selection can be decomposed into several components of selection that affect individuals at various stages during the life cycle (Christiansen and Frydenberg 1973) and that these episodes can provide important insights into mating systems (Emlen and Oring 1977), or ecological factors acting as agents of selection (Loehle and Pechmann 1988). Episodes of selection can also help evaluate threats and conservation issues (Stockwell et al. 2003). Additionally, much of the quantitative genetics theory commonly used in empirical studies focuses on individual episodes of selection (Arnold and Wade 1984a,b), so having the ability to examine the effects of selection at different episodes on the genome might be useful in linking theory to empirical work. Therefore, a method to detect the signature of each component of selection within a natural population would be an important addition to an evolutionary biologist’s toolkit.

Currently, three principal analytical methods are used to diagnose the effects of selection on the genome. First, quantitative traits can be mapped to specific loci using linkage mapping techniques. Quantitative trait locus mapping is very effective, but requires crossing specific parents, generating numerous offspring, and having a trait of interest to map. Second, genome-wide association studies can be used to correlate a specific trait (often disease related) with loci that differ between groups with different values of the trait (e.g., a group with diabetes compared to a group without; reviewed in Carlson et al. 2004). Finally, population genomics methods compare summary statistics describing allele frequencies, genetic diversity, and linkage disequilibrium between multiple populations of the same species to identify loci that lie outside of a specified significance threshold (Hohenlohe et al. 2010b). This method can be very powerful at detecting signatures of positive selection (e.g., Gagnaire et al. 2013; Hess et al. 2013), balancing selection (e.g., Reitzel et al. 2013), local adaptation (e.g., Hohenlohe et al. 2010a; Miller et al. 2012; Catchen et al. 2013a; Vincent et al. 2013), and selective sweeps (e.g., Boitard and Rocha 2013; Clement et al. 2013; Harris et al. 2013; Hubner et al. 2013; Rellstab et al. 2013). One shortcoming of comparing population genomics statistics between multiple populations is that such comparisons do not facilitate a diagnosis of the type of selection (e.g., sexual selection or viability selection) causing the pattern.

A complementary approach, which has not yet been applied on a whole-genome scale, is to measure the effects of selection at various stages in the life cycle of a population. At least four major types of selection occur during a typical life cycle. These different components can be isolated within a single generation using a cross-sectional study design (e.g., Christiansen and Frydenberg 1973; Christiansen et al. 1973), or by tracking a population over multiple generations in a longitudinal design (Bundgaard and Christiansen 1972; Clark and Feldman 1981; Clark et al. 1981; Anderson et al. 2014). Although a longitudinal design allows researchers to track allele frequencies over multiple generations, it is not a feasible experimental design for many organisms and is difficult to implement in studies of natural populations.

Selection components analysis can be used to decompose total selection into its parts in a variety of ways. For instance, some researchers have compared preobservation and postobservation components of selection (Prout 1965, 1969, 1971a,b), while others have examined mother–offspring combinations, allowing a subset of the male breeding population to be inferred and compared to a random sample of adult males containing both mated and unmated individuals (e.g., Christiansen and Frydenberg 1973; Nadeau and Baccus 1981). Allele frequencies of individuals at different life history stages were commonly compared in studies using allozyme markers (e.g., Christiansen and Frydenberg 1973, 1974; Christiansen et al. 1973; Nadeau and Baccus 1981; Heath et al. 1988; McDonald 1989), but these studies usually did not target enough loci to detect selection. Selection components analysis was also used to investigate the patterns of selection on entire chromosomes (e.g., Anderson 1969; Prout 1971a; Bundgaard and Christiansen 1972; Anderson et al. 1979; Clark and Feldman 1981; Clark et al. 1981; Curtsinger and Feldman 1980; Barbadilla et al. 1994), but chromosomes were too broad of a target and so only crude estimates of selection were detectable. However, with next-generation sequencing approaches it is now possible to identify large numbers of single nucleotide polymorphisms distributed across the entire genome, opening up the possibility to detect a genome-wide signature of selection components.

There is still much to learn about how selection affects the genome, and selection components analysis may be one solution. In this paper, we present findings from an individual-based simulation model that tests the application of existing population genomic approaches in the context of selection components analysis. We show that this approach holds promise for detecting genome-wide signatures of strong selection, at least in a best-case scenario. Additionally, this model allows us to make predictions about characteristics of populations that might benefit most from a selection components analysis approach.

## Methods

### Modeled sampling procedure

**Figure 1:**
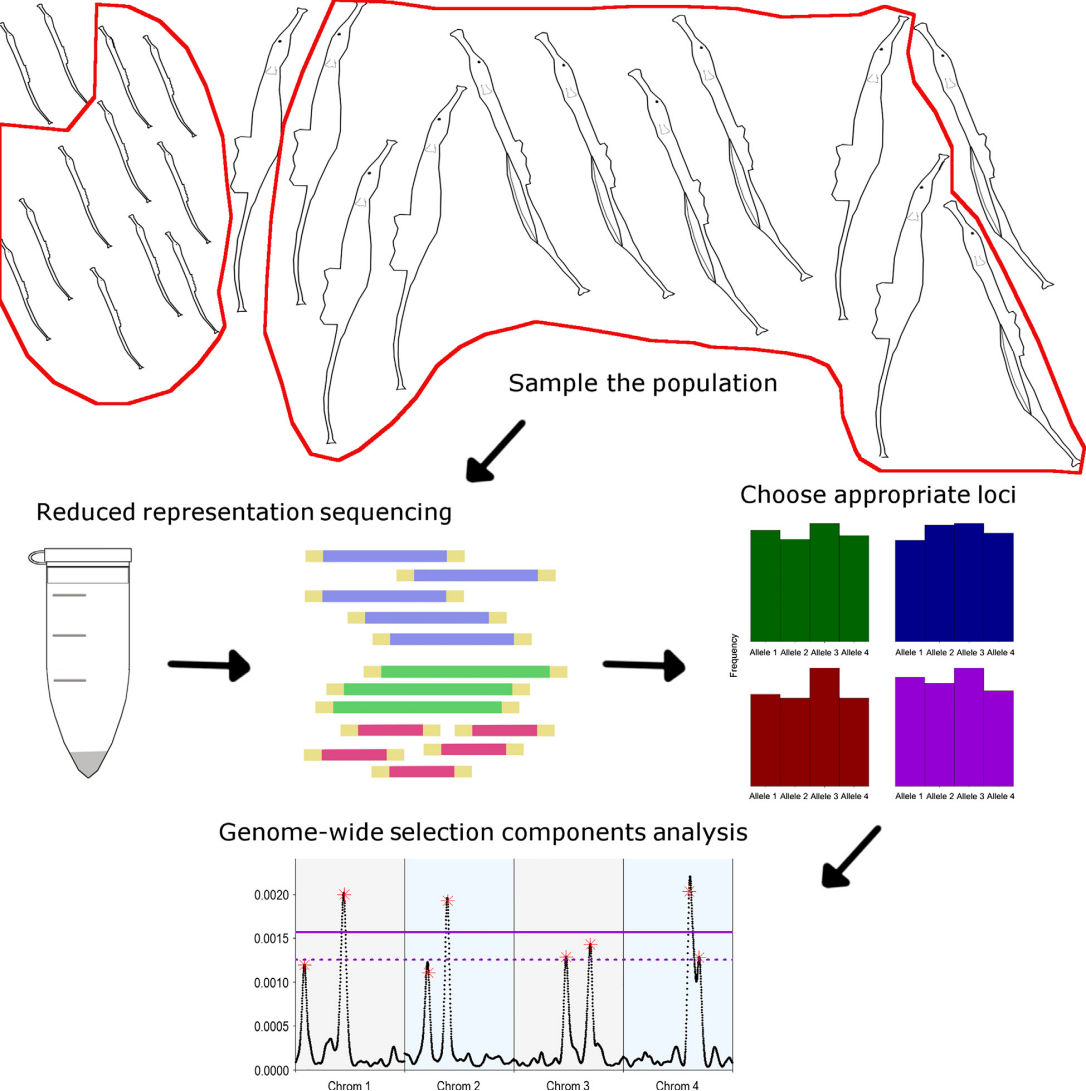
A schematic diagram of how to apply genome-wide selection components analysis in an empirical study. A population of some organism, for example, pipefish, is sampled so that DNA samples are obtained from roughly equal numbers of very young offspring and adults. Those DNA samples are then sequenced using a reduced representation sequencing method, such as RAD-sequencing. Loci with roughly even allele frequencies would then be selected for use in genome-wide selection components analysis, which will identify outlier loci that are putatively under selection.

This model, described in detail below, was designed to determine the power of an empirical selection components analysis. An empirical study would require a one-time collection of a population, including equal numbers of adults and offspring. The sampled individuals would then undergo some form of reduced representation sequencing, such as restriction-site-associated DNA sequencing (RAD-seq), to generate SNP data. From the genome-wide SNP data, loci with approximately uniform allele frequencies would be selected for genome-wide selection components analysis (Fig.1). From this analysis, we expect to detect only loci of large effect, as every population genomics study struggles to detect loci of small effect (Lewontin and Krakauer 1973; Beaumont and Nichols 1996).

In the implementation of our simulation model, which encapsulates a best-case scenario for this type of empirical study, we wished to model a population with genetic variation upon which selection could act. Thus, we modeled initial generations without sexual selection (i.e., with random mating). Even though a natural population would not typically make a single-generation transition from random mating to strong sexual selection, our approach uses this modeling convenience to simulate populations in a way that gives us control over levels of genetic variation and patterns of linkage disequilibrium independent of the strength of sexual selection. We chose to model a polygynous mating system (females mate once, males mate multiply), with sexual selection acting on the male trait, which is constrained by viability selection because this is a well-studied sexual selection framework in quantitative genetics (e.g., Lande 1981). Although other mating systems certainly exist and have strong sexual selection, we restricted our analysis to this natural selection and sexual selection trade-off for the scope of this paper.

### Model overview

The model was written in C++, and the source code is available on Dryad (doi: 10.5061/dryad.5k84d). We modeled a population with a carrying capacity of *N* individuals, each of which had *c* chromosomes with *m* markers (i.e., single nucleotide polymorphisms) and *q* quantitative trait loci. The quantitative trait loci additively determined, sometimes with added environmental noise, the phenotype of each individual. In a life cycle, individuals produced gametes, mated, and produced offspring. Females chose mates based on the encountered males’ phenotypes, putting sexual selection pressure on the male phenotype only. The male offspring then underwent viability selection on the same trait females used to choose a mate. Finally, the offspring matured into adults and replaced the previous generation.

The life cycle was repeated for a given number of initial generations, in which selection did not occur, to generate enough genetic variation upon which sexual selection could act. The initial generations were followed by one experimental generation, during which the population was randomly sampled and summary statistics calculated. Allele frequencies were also compared between adults and offspring (see below, “Sampling the population,” for more detail) using weighted *F*_ST_ values. We tested some of the parameters, such as the number of initial generations, to fine-tune the model so that we could simulate the best-case scenario for applying genome-wide selection components analysis.

Because we wanted to focus our attention on the types of markers that would be most informative in an empirical RAD-seq type of study, namely quantitative trait loci of large effect with moderate allele frequencies, our initial generations generated quantitative trait loci with relatively uniform distributions. This approach differs from previous work, which also used simulation models (e.g., Thornton et al. 2013), but instead focused on detecting rare alleles of moderate effect.

### Genetics of the population

The simulated organism was assumed to be diploid. Both neutral markers and quantitative trait loci were evenly distributed among chromosomes. The locations of quantitative trait loci were randomly chosen per chromosome per run of the model, unless otherwise stated. For instance, under the basic parameter combinations, each of the four chromosomes had 1000 marker loci and two quantitative trait loci, so that the total number of observed markers was 4000 and the total number of quantitative trait loci was eight. Although a suite of 4000 loci is a modest number of markers in the scheme of all loci identified in RAD-seq studies, most studies do typically restrict their analyses to several thousand loci. Therefore, we believe that 4000 markers is a reasonable number. The alleles for the quantitative trait loci were drawn from a normal distribution with a mean of zero and a standard deviation of 0.5. Each locus could have up to four alleles, and we started each simulation run with chromosome-wide genotypes for each chromosome. In other words, each run started with complete linkage disequilibrium within particular chromosomes. Linkage disequilibrium then decayed during the initial generations due to recombination, which occurred during the production of gametes in the form of *r* crossing-over events, where *r* was drawn from a Poisson distribution with a constant mean of 0.2. Each recombination event was randomly assigned a location between two marker loci. This approach allows the genome-wide level of linkage disequilibrium to be altered by merely changing the number of initial generations. No mutations occurred during the production of gametes, because the simulation runs consisted of so few generations that mutation would not be a major factor affecting allele frequencies. Phenotypes were calculated by summing across all alleles at all quantitative trait loci plus an added value, *e*, a random number from a normal distribution with a mean of 0 and a specified environmental standard deviation.

We tested different numbers of initial generations to see their effect on linkage disequilibrium and on the prospects for reliably detecting quantitative trait loci. For this analysis, we calculated pairwise linkage disequilibrium between 100 randomly chosen loci on each chromosome (i.e., all comparisons were from loci on the same chromosome). The pairwise linkage disequilibrium between randomly chosen locus A and locus B was calculated as follows. For each allele *A*_*i*_ and *B*_*j*_, *D* was calculated as *f*_*ij*_ *− p*_*i*_*q*_*j*_, where *f*_*ij*_ is the frequency of the *A*_*i*_ *B*_*j*_ haplotype, *p*_*i*_ is the frequency of allele *i,* and *q*_*j*_ is the frequency of allele *j*. *D*_max_ is the lesser of *p*_*i*_*q*_*j*_ or 1 − *p*_*i*_*q*_*j*_ when *D*_*ij*_ < 0 and is the minimum of (1 − *p*_*i*_)*q_*i*_ or *p*_i_*(1 − *q*_*i*_) when *D *>* *0. Finally, *D′* was evaluated as:

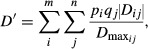


where *m* is the number of alleles at locus A and *n* is the number of alleles at locus B.

### Mating, production of gametes, and selection

**Figure 2:**
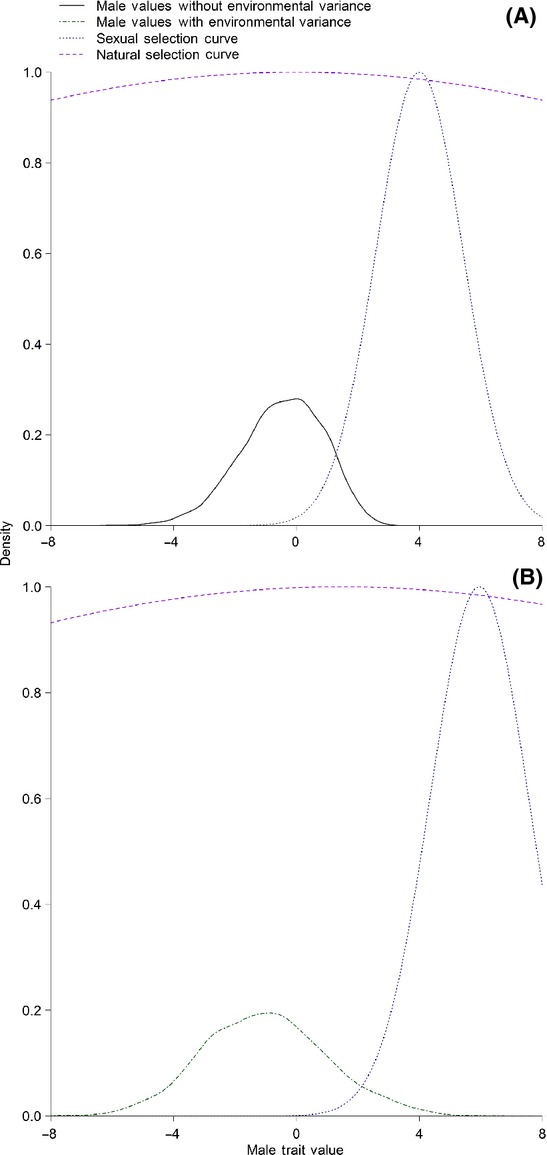
Representative distributions of male trait values under default parameters (A) and with an environmental variance of 1 (heritability = 0.5; B). Also shown are the uniform distributions based on the trait values and the preference optimum (sexual selection curve, *θ *= 4) and the viability selection optimum (natural selection curve, *θ *= 0) with default selection strengths (Table1).

In this model, each female mated with at most one male, and males were capable of mating with multiple females. Females randomly sampled 50 males in the population, and if they could not find an acceptable mate within those 50, they did not mate. We incorporated this cost of choosiness to add variability to the selection differentials in males. In our framework, males with identical trait values are not necessarily guaranteed the same number of matings. In the initial generations, females mated with the first male they encountered, so no sexual selection occurred. Sexual selection was introduced to the model during the experimental generation, after trait values were standardized to a mean of 0 and a standard deviation of 1. When mate choice was implemented, the probability that the female would mate with a given male was determined by a Gaussian-shaped function comparing the male’s phenotype, *z*, to a population-level female preference value, *θ*,

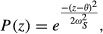


where 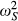 is the width of the selection surface (i.e., it determines the strength of selection). We set *θ* to an arbitrary value of 4 for all runs of the model. If a random number from a uniform distribution (0,1) was less than *P*(*z*), the female mated with that male and they produced four offspring. Therefore, the probability of mating for a male was determined first by whether a female encountered him and then by his trait value (*z*) relative to the population-level preference optimum (*θ *= 4). When selection was strong, few males possessed a trait value favored by the females (Fig.2).

When females found a mate and produced offspring, we simulated meiosis in the following way. For each chromosome pair, one of the mother’s two chromosomes was randomly chosen to be passed to the offspring. Before being passed to the zygote, recombination occurred. The number of recombination events on a given chromosome was a random number chosen from a Poisson distribution with a mean of 0.2. For each recombination event that occurred in a given mating event on a given chromosome, a randomly chosen chunk of one of the mother’s chromosomes was exchanged with the matching region from the mother’s other homologous chromosome, while maintaining the total size of each chromosome. The recombined chromosome was then passed to the zygote. A similar procedure was used for the father’s chromosomes, so recombination occurred in both sexes. This procedure realistically simulates the process of meiosis for a species in which crossing-over occurs at a similar rate in both sexes. The sex of each offspring was determined randomly, such that on average 50% of the offspring were female and 50% were male.

After the zygotes were produced, viability selection acted on the male offspring. Viability selection was implemented in the model merely to maintain variation in the male trait and to constrain sexual selection, and thus viability selection was a weak force in the model. This selection was implemented as the following Gaussian fitness surface with a given width, 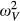:

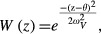


where *z* is an individual’s phenotype and *θ* is the optimum value (zero). Viability selection was implemented during both the initial generations and the experimental generations, although the strength of viability selection was weak 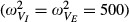. For each male, if a random number drawn from a uniform distribution (0,1) was less than *W*(*z*) for that individual, he survived to the next generation. As females did not express the trait, they were unaffected by viability selection. We implemented very weak viability selection, so that the majority of males survived the viability selection event (Fig.2A), even when environmental variance altered the distribution of male trait values (Fig.2B). After the viability selection event, offspring were randomly chosen to survive to adulthood, so that the number of surviving offspring was less than or equal to the carrying capacity.

### Sampling the population

Population demographic statistics were calculated for the entire population each generation. Some of the statistics calculated were population size, sex ratios, and mean trait values for males and females. Additive genetic and phenotypic variances were calculated from the distribution of values in all adults. Heritability was calculated as the additive genetic variance divided by the phenotypic variance and was therefore always 1 whenever there was no environmental variation added to the trait. “Long-distance” linkage disequilibrium was calculated for randomly selected loci throughout the genome as described above (see “Genetics of the population”), and the same equations were used to calculate pairwise linkage disequilibrium between neighboring polymorphic loci. Mating differentials were calculated as the covariance between standardized trait values and relative mating success (Jones 2009).

During the experimental generation, the population was randomly sampled after mating occurred and offspring were produced, but before the offspring experienced viability selection. As females all produced the same number of offspring (4) and meiotic drive was not included in the model, this sampling strategy captured the effects of sexual selection on allele frequencies. Both parents and offspring were sampled without replacement, and genealogical relationships were assumed to be unknown. Summary statistics, including allele frequencies and observed and expected heterozygosities, were calculated for adults, offspring, and the total population. Expected heterozygosity, *H*_*E*_, was calculated as 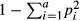 for each locus with *a* alleles. We then compared allele frequencies in adults and offspring using the Nei (1986) *F*_ST_ calculation:

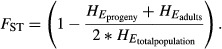


*F*_ST_ values were weighted using a kernel-smoothing moving average, which incorporates the contribution of nearby values to the *F*_ST_ for each locus. Specifically, each polymorphic locus, *k*, was weighted by the *F*_ST_ values at each marker position, *d*, within the sliding window region in each direction, using the Gaussian function:

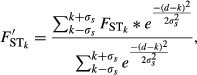


where *σ*_*s*_ is the width of the sliding window region in each direction (Hohenlohe et al. 2010a).

Much work in the field of population genetics has been dedicated to detecting *F*_ST_ outliers, beginning with Lewontin and Krakauer (1973). They proposed the idea that neutral markers all experience the same background selection, drift, and other demographic factors, so any loci with statistics such as *F*_ST_ lying outside of the distribution of the other loci are likely experiencing selection (Lewontin and Krakauer 1973). Improvements on the original method have been suggested, such as weighting *F*_ST_ values by heterozygosity (Beaumont and Nichols 1996) and using Bayesian methods (Beaumont and Balding 2004). Other studies have tested the importance of the neutral distribution, by comparing null models to the dataset (e.g., Foll and Gaggiotti 2008; Lotterhos and Whitlock 2014). Indeed, other work has shown that increased neutral variance in *F*_ST_ values leads to high rates of false positives (Bierne et al. 2013). Despite the acknowledged importance of the distributions of *F*_ST_ values in determining outliers, the distribution of the smoothed *F*_ST_ values used commonly in modern population genomics studies is unknown, and the neutral distributions are often not mentioned in population genomics analyses. We were therefore interested in evaluating different common methods for determining significance of our outlier summary statistics.

We implemented three methods of determining cutoffs, two of which are commonly used approaches in the literature. First, we calculated *P*-values for each 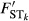 statistic at locus *k* using the *χ*^2^ distribution, as *F*_ST_ values are known to have a *χ*^2^ distribution in a neutral model (Workman and Niswander 1970; Lewontin and Krakauer 1973; Weir and Cockerham 1978; Beaumont and Nichols 1996). Specifically, the transformation 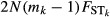 is the *χ*^2^ statistic, where *m* is the number of alleles at locus *k* in *N* individuals, with (*m − 1*)(*n* − 1) degrees of freedom, where *n* is the number of subgroups within a population (Workman and Niswander 1970). The Benjamini and Hochberg (1995) false discovery rate was then calculated to establish a cutoff value by ranking all of the *P*-values from smallest to largest. For each *P-*value, its relative rank (its order in the sorted list of *P-*values divided by the total number of *P-*values) was multiplied by the significance value, 0.05. The largest *P*-value that was less than or equal to this weighted rank was the false discovery rate significance threshold. Second, we implemented the bootstrapping algorithm used by the software package Stacks (Catchen et al. 2011, 2013b), a common population genomics bioinformatics program, re-sampling the genome 10,000 times. This algorithm re-weights the weighted 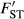 values using the kernel-smoothing approach described above, but the nucleotide positions (*d*) are randomly chosen loci from anywhere in the genome, rather than the neighboring nucleotides. Confidence intervals were then calculated from the distribution of the 10,000 bootstrapped *F*st′ values in the same way as described below for the genome-wide confidence intervals. Finally, we determined the genome-wide distribution of 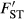 values and calculated confidence intervals. Although *F*_ST_ values have a *χ*^2^ distribution (Workman and Niswander 1970; Lewontin and Krakauer 1973; Beaumont and Nichols 1996; Lotterhos and Whitlock 2014), the distribution of smoothed *F*st′ values is unknown and appears to only approximate a *χ*^2^ distribution. Therefore, rather than calculating confidence intervals from a *χ*^2^ distribution, we chose to use a Gaussian confidence interval, which is based on two basic descriptors of the 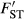 distribution: the mean and the variance. To calculate the confidence intervals, the mean 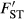 and the variance and standard deviation in 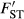 values were calculated across all sampled loci on all chromosomes. Genome-wide confidence intervals were then calculated as the mean 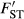 value plus the appropriate value from the cumulative normal distribution function multiplied by the standard deviation of 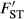 values. For example, the 95% genome-wide confidence interval is calculated as follows:

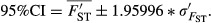


We present the 95% and 99% confidence intervals, as those are two significance thresholds commonly used in biology, although other confidence intervals certainly could be used instead. After determining these various cutoff values, each peak in *F*st′ value was detected and the value compared to the cutoffs. If the peak was above the cutoff value, and if a known quantitative trait locus was within *x* marker loci of the peak, then it was counted as a “real” peak. If the peak was above the cutoff value but a quantitative trait locus was not within *x* marker loci, then it was counted as a “spurious” peak. The average and standard errors of both the number of real and spurious peaks were calculated. The average number of real peaks detected compared to the overall number of quantitative trait loci reflected the amount of type II error in the analysis. In contrast, the average number of spurious peaks indicated the extent to which type I error occurred (i.e., the frequency of false positives). To test various peak widths, we varied *x* and tested values from 1 to 100 loci.

### Testing parameter combinations

The default parameters from the model are in Table1. To address whether genome-wide selection components analysis could be used in empirical studies of natural populations, we focused on the effects of sample size, strength of sexual selection, and environmental variance in the focal trait. We also assessed the effects of the architecture of marker loci (covarying the number of marker loci and number of chromosomes), population size, and the number of quantitative trait loci underlying the trait on this type of selection components analysis. We tested many of these parameters in combination. The pairwise parameter combinations tested included sample size and carrying capacity; the number of markers and the number of chromosomes; the number of markers and sample size; the strength of sexual selection and number of quantitative trait loci; the strength of selection and linkage disequilibrium; environmental variation and the number of quantitative trait loci; and environmental variation and linkage disequilibrium.

**Table 1:** The baseline parameters for running the simulation model. Selection variances refer to the Gaussian selection surface width. “Initial” refers to the width during the initial generations before sexual selection was imposed, and “Experimental” refers to the width during the subsequent generation during which the population was sampled

Parameter	Starting value
Carrying capacity	5000
Sample size (adults)	4000
Sample size (offspring)	4000
Number of markers per chromosome	1000
Number of QTL per chromosome	2
Number of chromosomes	4
Initial mate choice strength 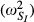	Random mating
Experimental mate choice strength 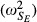	2
Initial viability selection strength 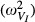	500
Experimental viability selection strength 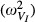	500
Environmental variance	0
Number of populations	1

## Results

### Peak detection

**Figure 3:**
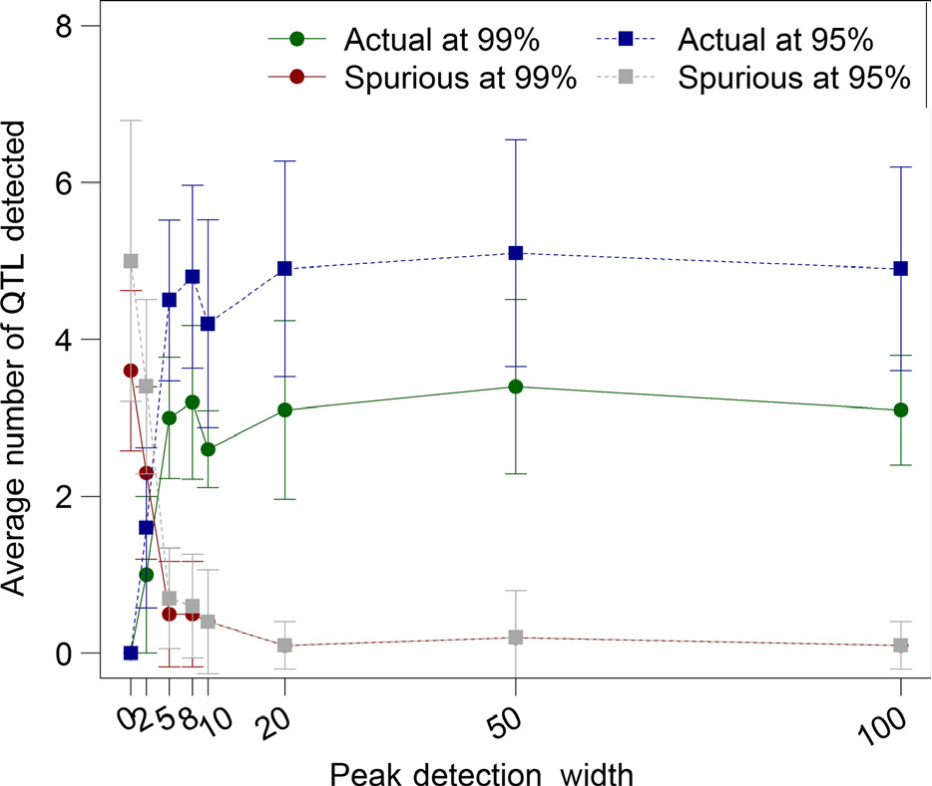
The effect of peak detection width on average number of quantitative trait loci detected. When a peak was identified, it was either designated “actual” or “spurious” based on whether there was a quantitative trait locus *x* markers away. The value of *x* is the “peak detection width,” represented on the *x-*axis here. To test peak detection width, the model was run with default parameters (Table1), with 10 replicates for each peak detection width tested. Bars are the standard error of the mean.

We explored how the peak detection width, or the distance from an actual quantitative trait locus required to call a peak a “real” peak as opposed to a spurious peak, affected the results. All of the values we tested beyond 2 loci resulted in equivalent results (Fig.3). We used a peak detection width of 50 loci for all of the other runs of the model.

### Replication

To determine whether running the model with only ten replicates would negatively impact detection rates, we ran the default parameters (Table1) with both 10 replicates and 100 replicates. We found qualitatively similar detection rates (Table2), so to conserve time we ran the model for 10 replicates for all parameter combinations.

**Table 2:** A comparison of running the model with default parameters (Table1) and with either 100 replicates or 10 replicates. Here we display both the 99% and 95% cutoffs as determined by the genome-wide confidence interval and the bootstrapped confidence interval. We also present the 95% false discovery rate. Running 100 replicates instead of 10 replicates did not significantly increase the average number of real peaks (“real”) or decrease the average number of spurious peaks (“spurious”)

Detection method	Significance level	Number of replicates	Real ± SE	Spurious ± SE
Genome-wide confidence interval	99%	100	3.300 ± 1.22	0.000 ± 0.00
		10	3.800 ± 1.25	0.100 ± 0.30
	95%	100	4.970 ± 1.45	0.000 ± 0.00
		10	5.200 ± 1.33	0.100 ± 0.30
Bootstrapped confidence interval	99%	100	0.948 ± 0.85	0.113 ± 0.84
		10	0.875 ± 0.79	0.088 ± 0.84
	95%	100	0.948 ± 0.08	0.145 ± 0.15
		10	0.875 ± 0.30	0.100 ± 0.09
False discovery rate	95%	100	0.169 ± 0.36	0.580 ± 1.46
		10	0.200 ± 0.40	0.838 ± 1.65

### Determining significance

Our three methods of choosing cutoff values for determining whether a peak was significant showed strikingly different patterns. The false discovery rate was highly unpredictable, such that in some cases nearly every locus was significant, and at other times nearly none of the loci were significant, when the parameters remained constant. This unpredictability is reflected in the standard errors of the mean number of spurious peaks detected and especially in the mean proportion of peaks detected (Table3). Additionally, the false discovery rate detected very few actual peaks. The bootstrapped confidence intervals were better than the false discovery rate and detected a high number of real peaks, but consistently detected spurious peaks (Table3). In contrast, the genome-wide confidence intervals were more conservative in the number of peaks detected, but consistently detected over 30% of the real peaks, and importantly very rarely detected spurious peaks (Table3). Thus, for the rest of our results, significance was determined by 95% or 99% genome-wide confidence intervals. It is important to note that rarely were all quantitative trait loci detected, largely because quantitative trait loci of small effect tended not to show strong signatures of selection. The best detection rate achieved was 92.5% of the quantitative trait loci, with a spurious detection rate of 0, but more commonly a “good” detection rate was 50-70%.

**Table 3:** The reliability of three methods to determine significance cutoff thresholds for weighted *F*_ST_ values was compared using the mean proportion of actual peaks detected and the mean number of spurious peaks detected and their standard errors. These means were generated by running the model with its default parameters (Table1) in 10 replicates, each of which had 200 initial generations where no sampling occurred, followed by one experimental generation. Allele frequencies and *F*_ST_ measures were calculated between adults and offspring. Viability selection was weak during both the initial and the experimental generations 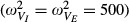, and strong sexual selection was introduced at the start of the experimental generations 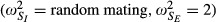

	Mean proportion of actual peaks detected	SE	Mean number of spurious peaks detected	SE
99% Genome-wide CI	0.3475	0.0245	0.1000	0.0428
95% Genome-wide CI	0.4925	0.0318	0.1000	0.0428
99% Bootstrapped CI	0.8000	0.0404	1.1400	0.1874
95% Bootstrapped CI	0.8075	0.0395	1.5600	0.2063
False discovery rate	0.1525	0.0455	4.0400	1.5367

### Linkage disequilibrium

**Figure 4:**
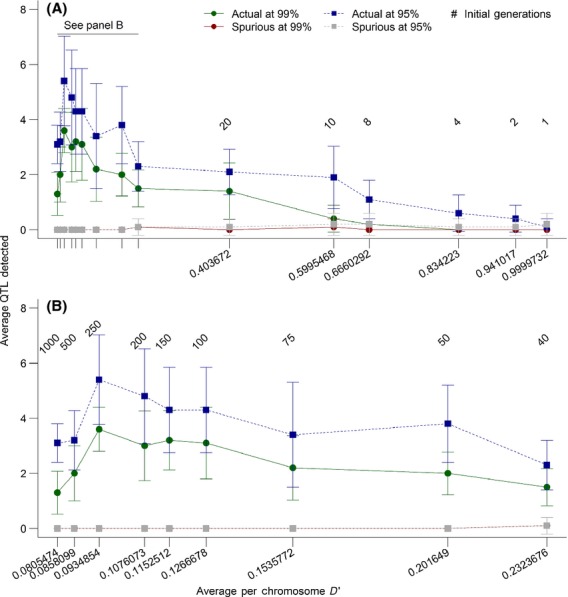
The effect of linkage disequilibrium (determined by the number of initial generations) on the percentage of quantitative trait loci accurately detected and the number of spurious loci called as significant by 99% and 95% genome-wide confidence intervals for weighted *F*_ST_ values. The measure of linkage disequilibrium presented here is *D′* calculated as a pairwise measure between 100 loci randomly selected from each chromosome and averaged across chromosomes and replicates. Linkage disequilibrium was calculated in the final initial generation, and the number of initial generations was varied to change linkage disequilibrium (initial generation numbers are presented above the points on the graphs). Panel A shows all permutations of the number of initial generations (1, 2, 4, 8, 10, 20, 40, 50, 75, 100, 150, 200, 250, 500, and 1000). Panel B presents a close-up view of generations 40, 50, 75, 100, 150, 200, 250, 500, and 1000 to highlight the changes that occur at low levels of linkage disequilibrium (below 0.3). The model was run with the parameters presented in Table1. Values presented here are averages from 10 replicates. Bars are the standard error of the mean.

The number of initial generations determined the degree of linkage disequilibrium present when sexual selection was introduced in the experimental generations. We measured long-distance linkage disequilibrium per chromosome (*D′*) at the end of the initial generations (see Methods: Genetics of the population). The number of initial generations tested varied from 1 to 1000 to examine how linkage disequilibrium affected our ability to detect selection. We found that after 200 initial generations linkage disequilibrium was 0.1007 and that detection rates appeared to peak at this level of linkage disequilibrium (Fig.4). We chose to use 200 generations in the rest of our permutations of the model to present the best-case scenario for our genome-wide selection components analysis approach.

**Figure 5:**
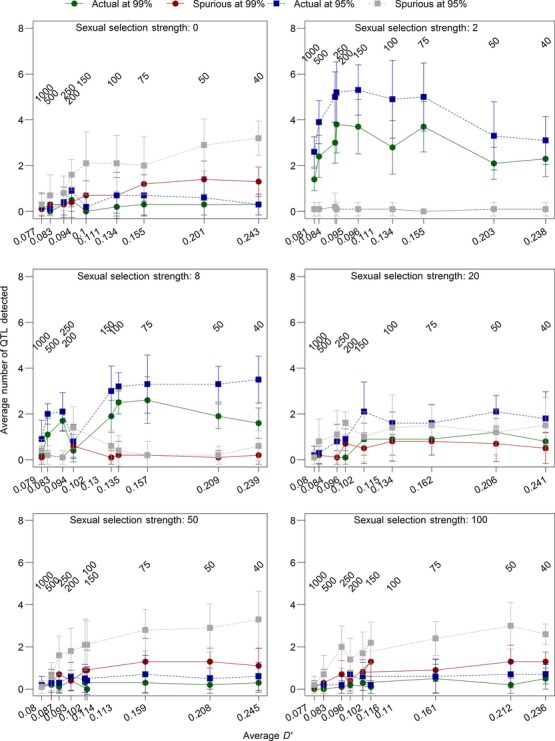
The effects of linkage disequilibrium and sexual selection on detection rates. The measure of linkage disequilibrium is *D′*, a pairwise measure of linkage disequilibrium between 100 loci randomly selected from each chromosome and averaged across chromosomes and replicates, calculated in the final initial generation. The number of initial generations was varied to change linkage disequilibrium. Because most of the effects on detection rates occurred at low levels of linkage disequilibrium (below 0.3; Fig.3), we restricted analysis to those measures, which represent 40, 50, 75, 100, 150, 200, 250, 500, and 1000 initial generations. The number of initial generations is presented above the points, and *D′* is presented on the *x-*axis. The model was run with default parameters (Table1), except for the altered number of initial generations and values of sexual selection in the experimental generations. Values presented are averages from 10 replicates with bars showing the standard error of the mean.

**Figure 6:**
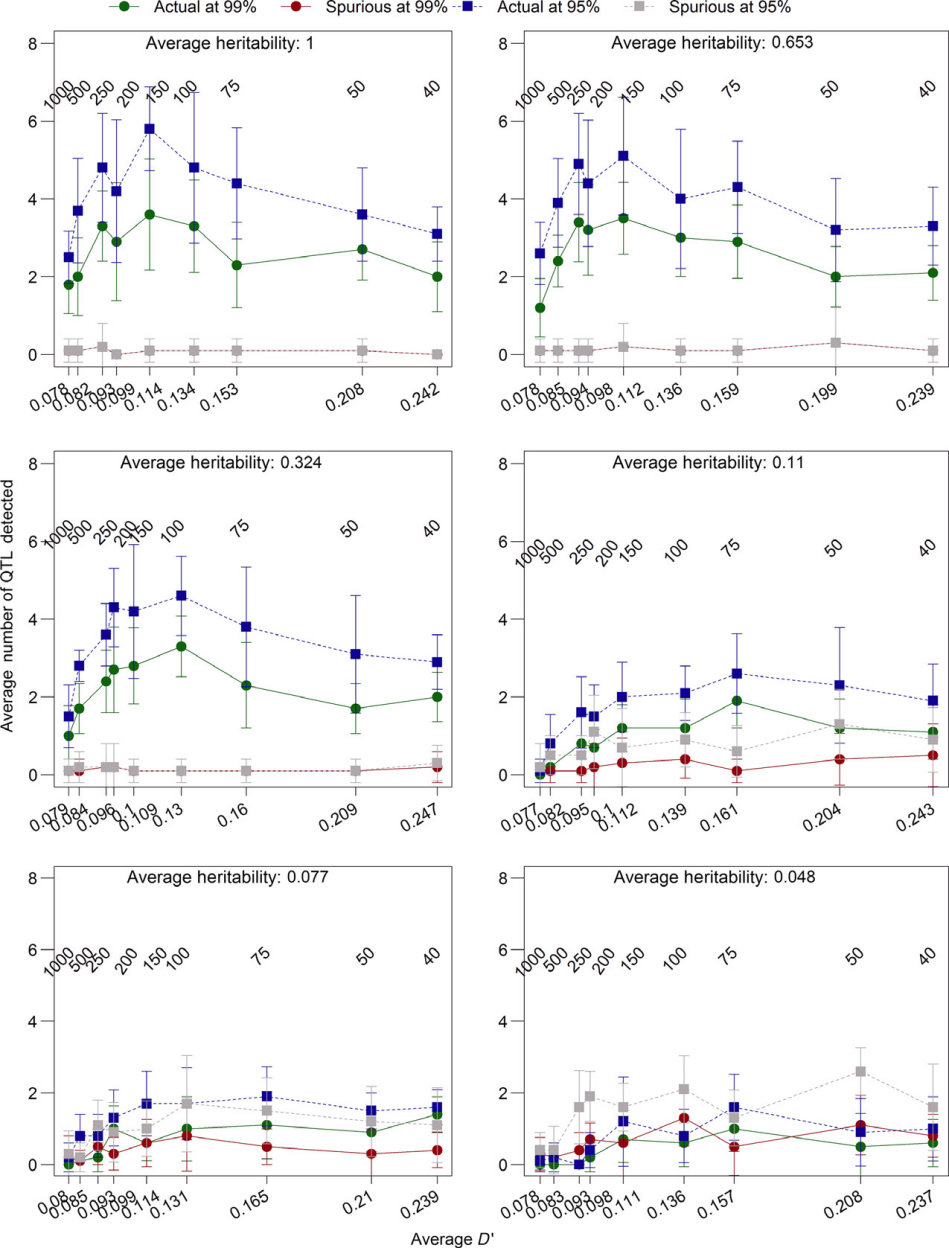
The effect of linkage disequilibrium and environmental variation on detection rates. Linkage disequilibrium, *D′*, was calculated as a pairwise measure between 100 randomly selected loci from each chromosome and averaged across chromosomes in the final initial generation. The number of initial generations is presented above the points, and *D′* is on the *x*-axis. We ran the model with default parameters (Table1), except for the number of initial generations and environmental variance. We present the average heritabilities generated by various environmental variance settings. Values shown are the means of 10 replicates with standard error bars.

We also explored the effects of linkage disequilibrium on the detection rate when sexual selection strength was varied (Fig.5) and when environmental variation in the trait was introduced (Fig.6). In both cases, there was not a large amount of variation in detection rates with different levels of linkage disequilibrium. Very low linkage disequilibrium (0.077–0.078, resulting from 1000 initial generations; Figs.6) resulted in reduced detection rates in both cases, as did high levels of linkage disequilibrium (≥0.2, resulting from 50 initial generations or fewer; Figs.6). This further solidifies our choice of 200 initial generations as providing us with a best-case scenario for testing genome-wide selection components analysis.

### The effects of population size and sample size

**Figure 7:**
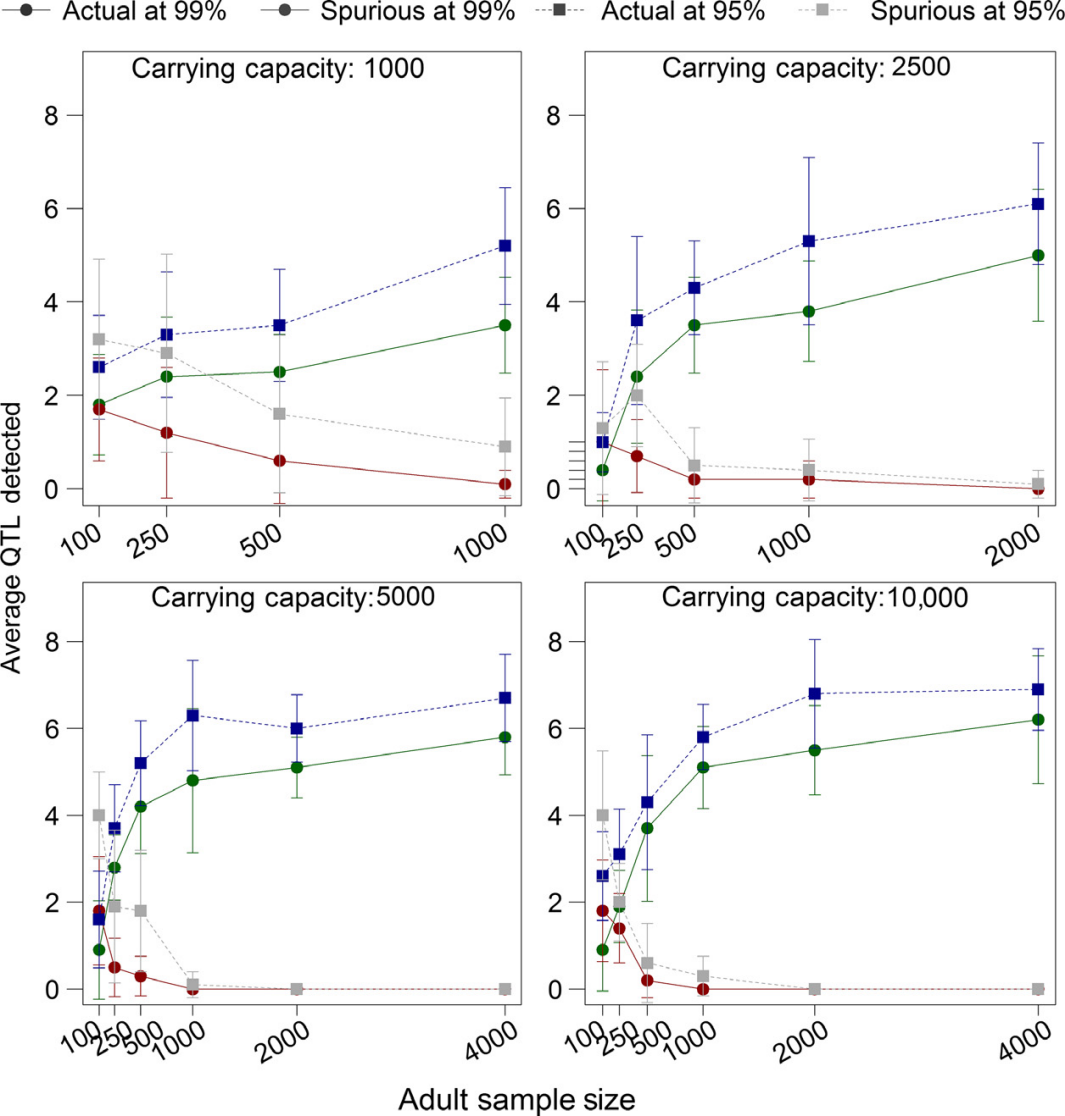
The effect of population size and sample size on detection rates. For each of 10 replicates, the model ran for 200 initial generations followed by one experimental generation. The number of real and spurious peaks detected was averaged across all ten replicates. The carrying capacity and sample size were constant throughout all initial and experimental generations, and the same number of adults and offspring was sampled in the experimental generation. All other parameters were the default parameters shown in Table1. Bars show the standard error of the mean.

Population genetics theory predicts that selection will have a stronger effect in larger populations due to a reduction in the effects of drift (Hartl and Clark 2007). Thus, we tested how well our selection components analysis detected selection in populations of varying sizes (1000, 2500, 5000, and 10,000) with different sample sizes (100, 250, 500, 1000, 2000, and 4000). Sample size and population size both impacted the detection rates, and they appeared to have an interactive effect. The minimum sample size tested, 100 adults and 100 offspring, had a high average number of spurious peaks detected (but this number was still below 1, suggesting that on average fewer than one spurious peak was detected) and a low proportion of peaks detected (only 18.5% at the 99% confidence level, which means that between 1 and 2 of the eight actual quantitative trait loci were detected; Fig.7). This pattern was consistent across population sizes. In other words, regardless of the actual population size, a sample of 100 adults and 100 offspring was barely adequate to detect any quantitative trait loci. However, increasing the sample size improved detection rates dramatically, especially in larger populations. As the population size increased, the mean number of spurious loci fell below the mean detection rate (Fig.7). Large sample sizes alone improved detection rates, but the combination of a large sample with a large population led to high detection rates as well as very low numbers of spurious loci detected.

### The effects of the number of neutral markers

**Figure 8:**
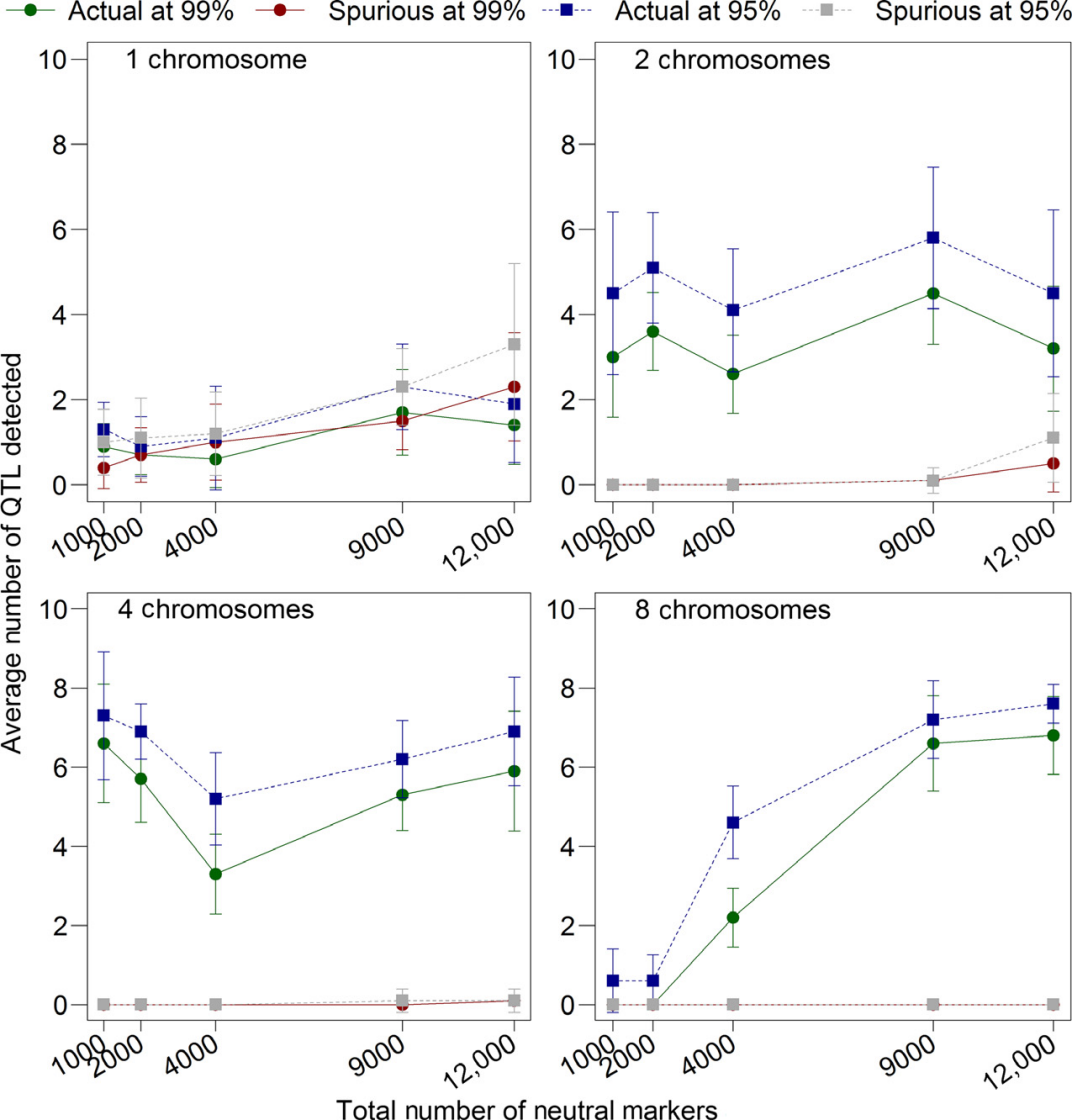
The effect of the number of neutral markers and number of chromosomes on detection rates. These data represent averages from ten replicates, each of which had a carrying capacity of 5000, adult and offspring sample sizes of 4000 each, and ran for an initial 200 generations followed by one experimental generation. A constant number of 8 quantitative trait loci were distributed equally across the chromosomes (so with 4 chromosomes, there were 2 quantitative trait loci on each, but the location of each quantitative trait locus on the chromosome was randomly chosen). Other than the number of chromosomes and the number of neutral markers, all other parameters were set to the defaults (Table1). Bars indicate the standard error of the mean.

In this model, the total number of neutral markers could be manipulated by changing the number of chromosomes, changing the number of markers per chromosome, or changing both. We investigated the interaction between the total number of markers (1000, 2000, 4000, and 9000) and chromosome number (1, 2, 4, and 8) on the detection of real and spurious peaks, and two major patterns emerged. First, regardless of how many chromosomes among which the loci were distributed, having more marker loci increased the average proportion of real peaks detected, but also slightly increased the number of spurious loci detected. The number of chromosomes seemed to determine the magnitude of the increase in spurious peaks. Low numbers of neutral markers (1000 and 2000) consistently had low detection rates (Fig.8). However, with the markers distributed across many chromosomes, the detection rate increased dramatically once there were more than 2000 markers. Indeed, with 12,000 markers spread evenly across 8 chromosomes, the quantitative trait locus detection rate was 87.3% at the 99% confidence level and 94.3% at the 95% confidence level, which are some of the highest values we recorded. The average 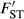 value for runs with 8 chromosomes was lower than the average 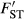 in runs with fewer chromosomes (e.g., with 9000 total neutral loci and eight quantitative trait loci, mean 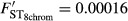 and mean 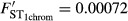 ). Essentially no spurious peaks were detected (Fig.8) under these parameter combinations. This pattern may be due to the fact that the quantitative trait loci were equally distributed among chromosomes, so with 8 chromosomes and 8 quantitative trait loci, there was exactly 1 quantitative trait locus on each chromosome.

### The effects of sample size and number of neutral markers

**Figure 9:**
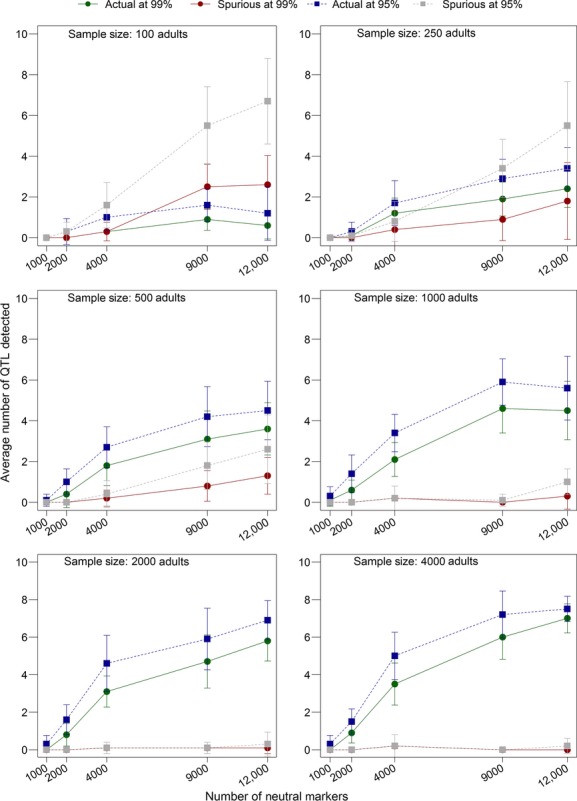
The effect of sample size and the number of neutral markers on detection rates. Each panel is a different sample size. The offspring sample size was set to be equal to adult sample size in each case. The neutral markers were distributed evenly across four chromosomes, each of which contained two quantitative trait loci. Besides sample size and the number of neutral markers, all other parameters were set to the defaults (Table1). The values presented here are means from 10 replicates with standard error bars.

As the number of neutral markers in our model represents the number of sampled markers in an empirical study, we explored how varying adult sample size and the number of neutral markers affected our results. We found a significant increase in the number of real quantitative trait loci detected when both sample size and the number of neutral markers were increased. When sample size was small (100 or 250 adults), we found an increase in the number of spurious markers detected with increasing number of neutral markers (Fig.9). This suggested that more peaks were likely to be detected, whether they were spurious or not, with an increased number of neutral markers, and that increasing the adult sample size allowed real peaks to be detected, as opposed to spurious peaks. This may be due to the fact that having more neutral markers diluted the effect of outlier *F*st′ values on the mean background *F*st′ values, allowing outliers to be even more differentiated from the background when there were more neutral markers.

### The effects of number of quantitative trait loci and strength of selection

**Figure 10:**
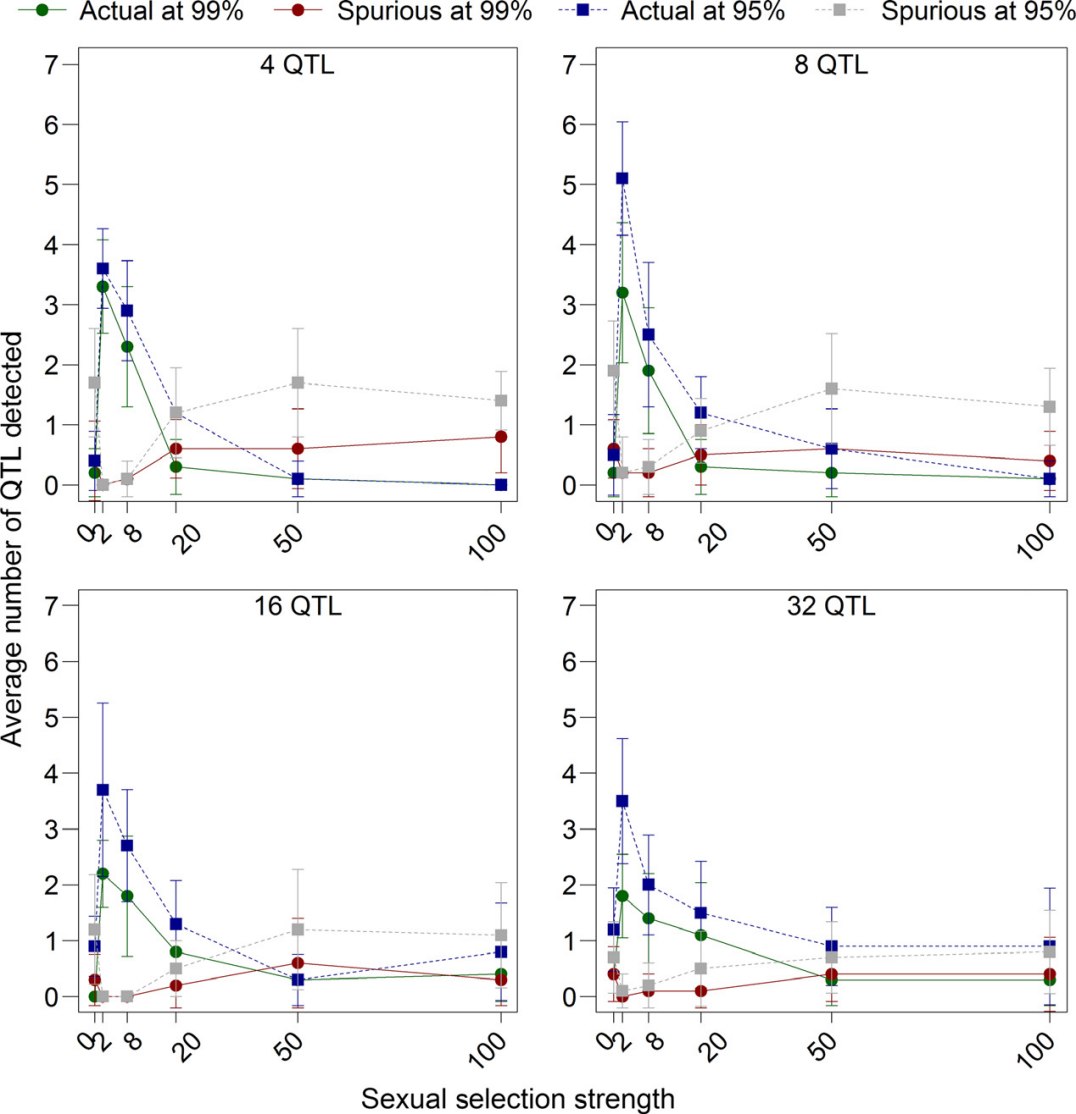
The effect of sexual selection strength and the number of quantitative trait loci on detection rates. We tested random mating and female choice with selection surface widths 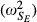 of 2, 8, 20, 50, and 100 during the experimental generation. There was random mating during the initial 200 generations, and these selection strengths were implemented during the experimental generation. The quantitative trait loci were distributed equally among the chromosomes, and the total number of quantitative trait loci tested was 4, 8, 16, and 32. These data are averages and standard errors from the experimental generation of 10 replicates.

The number of quantitative trait loci had a strong effect on the ability to detect real peaks, as we had predicted. The total strength of selection was distributed among all of the quantitative trait loci, so with fewer quantitative trait loci, each locus received a greater portion of the total selection. We tested sexual selection surface widths in the experimental generations 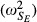 of 2, 8, 20, 50, 100, and 500 acting on a total of 4, 8, 16, and 32 quantitative trait loci distributed equally on 4 chromosomes. We also included a test with random mating in the experimental generation for comparison. Selection strength was greatest at 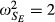 in each case (male *m′* ≈ 1.2). The number of quantitative trait loci had a large effect on the detection of selection: When strong selection was acting on a total of 32 quantitative trait loci, only 6.5% real peaks were detected at the 99% confidence level (∼2 of the quantitative trait loci), whereas with only 4 quantitative trait loci, 89.5% were detected on average (Fig.10). The improvement in detection rates with few quantitative trait loci when selection was strong came with a cost when selection was weak: The number of spurious peaks detected at low selection strengths and few quantitative trait loci was higher than the number of peaks detected at low selection strengths but many quantitative trait loci (Fig.10). Overall, these results suggested that accurately and reliably detecting selection required that selection acted strongly on phenotypes that were mainly determined by a few quantitative trait loci of major effect. Although this result was unsurprising, as it is well established that population genetics techniques can only detect loci of major effect (Lewontin and Krakauer 1973; Beaumont and Nichols 1996), it is worth noting that our within-population approach conforms to population genetics expectations.

### The effects of environmental variation

**Figure 11:**
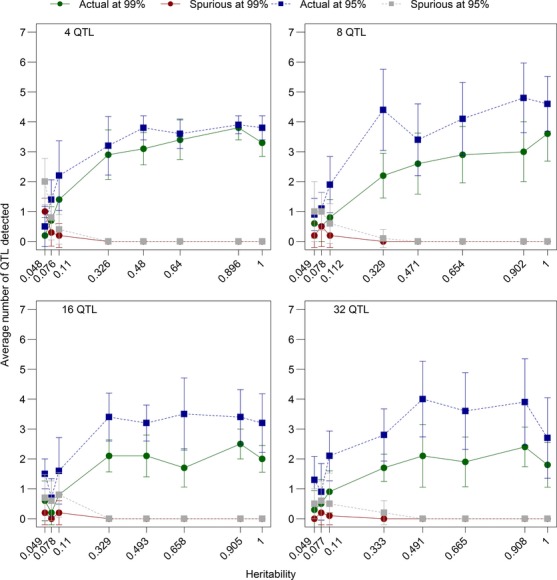
The effects of environmental variation on the ability to detect quantitative trait loci using an outlier *F*_ST_ approach. Environmental variation was implemented by drawing a number from a normal distribution with variances of 0, 0.1, 0.5, 1, 2, 8, 12, and 20 and adding that value to the phenotype of the individual. We calculated the average heritability from all 10 replicates for each environmental variance and present those values on the x-axis, rather than the environmental variances. The environmental variances were tested with 4, 8, 16, and 32 total quantitative trait loci, which were distributed equally among the chromosomes. All other parameters were set to the defaults (Table1). Error bars are the standard error of the mean.

The phenotype of an individual was determined by two components: the genotype derived from the quantitative trait loci and environmental effects. We tested how adding environmental variation to individuals’ phenotypes affected the reliability of our genome-wide selection components analysis by changing the environmental variance from zero (0, 0.1, 0.5, 1, 2, 8, 12, and 20) and the number of quantitative trait loci underlying the phenotype (4, 8, 16, and 32). Adding a small amount of environmental variance (0.1 or 0.5) did not have a large effect on our ability to detect quantitative trait loci under selection and even led to a slight increase in the proportion of real peaks detected (Fig.11). Once the environmental variance reached values greater than 1, the ability to detect loci under selection declined and the number of spurious loci detected increased (Fig.11). However, the variance in male trait values without added environmental variance was typically between 0 and 1 in this model, so adding a value of up to 20 to the phenotype may not be biologically relevant. Adding perhaps more relevant values (0.1, 0.5, 1, and 2) did not substantially alter the ability to detect selection (Fig.1).

The number of quantitative trait loci underlying the trait also affected the prospects for detecting selection but buffered the effects of environmental variation; when 32 quantitative trait loci affected the trait, the proportion of quantitative trait loci detected was consistently below 20%, but did not decline significantly with added amounts of environmental variation, and the number of spurious loci detected remained near zero (Fig.1). So although environmental variance added noise to the data, especially when many quantitative trait loci affected the trait of interest, it was still possible to detect some loci under selection even with low heritabilities. Our ability to detect a small subset of quantitative trait loci was likely due to random chance placing a spurious locus near a quantitative trait locus, so it is unclear how these detection rates would translate into empirical studies in natural populations.

### Comparing multiple populations

**Figure 12:**
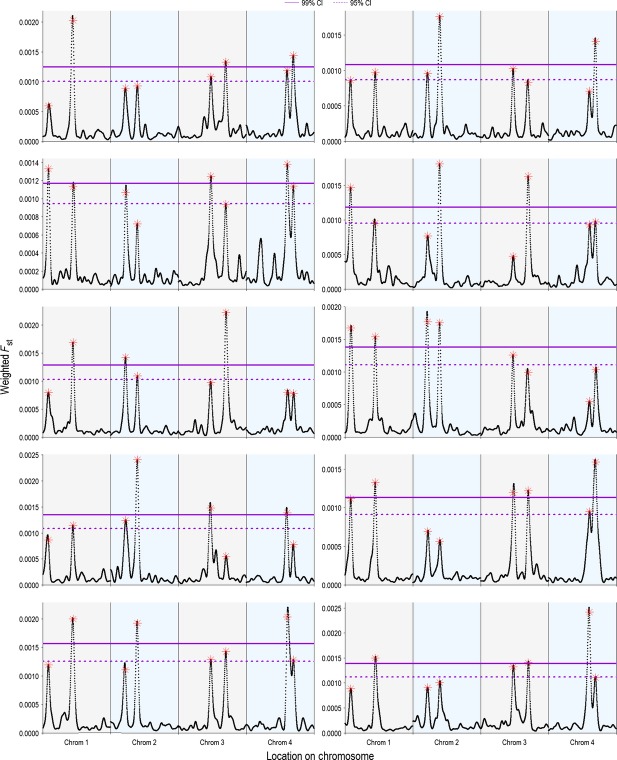
Sampling multiple populations can improve the detection of real quantitative trait loci, if the same loci underlie the trait affected by selection. The model was run with 10 replicates, but in each replicate the 8 same quantitative trait loci were designated, rather than being randomly assigned. Thus, each replicate was essentially another population with the same loci under selection, but without gene flow between populations. The model was run with the default parameters (Table1). Comparing the significant weighted *F*_ST_ values uncovered in each population, it is obvious that the peaks that reappear in each population are the quantitative trait loci (whose locations are designated by red asterisks). The four chromosomal regions are delineated by different colored backgrounds, and we show two genome-wide confidence intervals (99% and 95%) on the graph.

We ran the model on multiple populations that all had the same quantitative trait loci, but that experienced no gene flow, to determine whether comparing even distant populations of the same species might help improve the detection of quantitative trait loci. We found that there was no increase in the reliability of detection (within a population, the average number of real and spurious loci remained the same). However, as predicted, the spurious loci differed between populations, allowing peaks that were at consistent loci to be identified as “real” loci (Fig.12).

**Figure 13:**
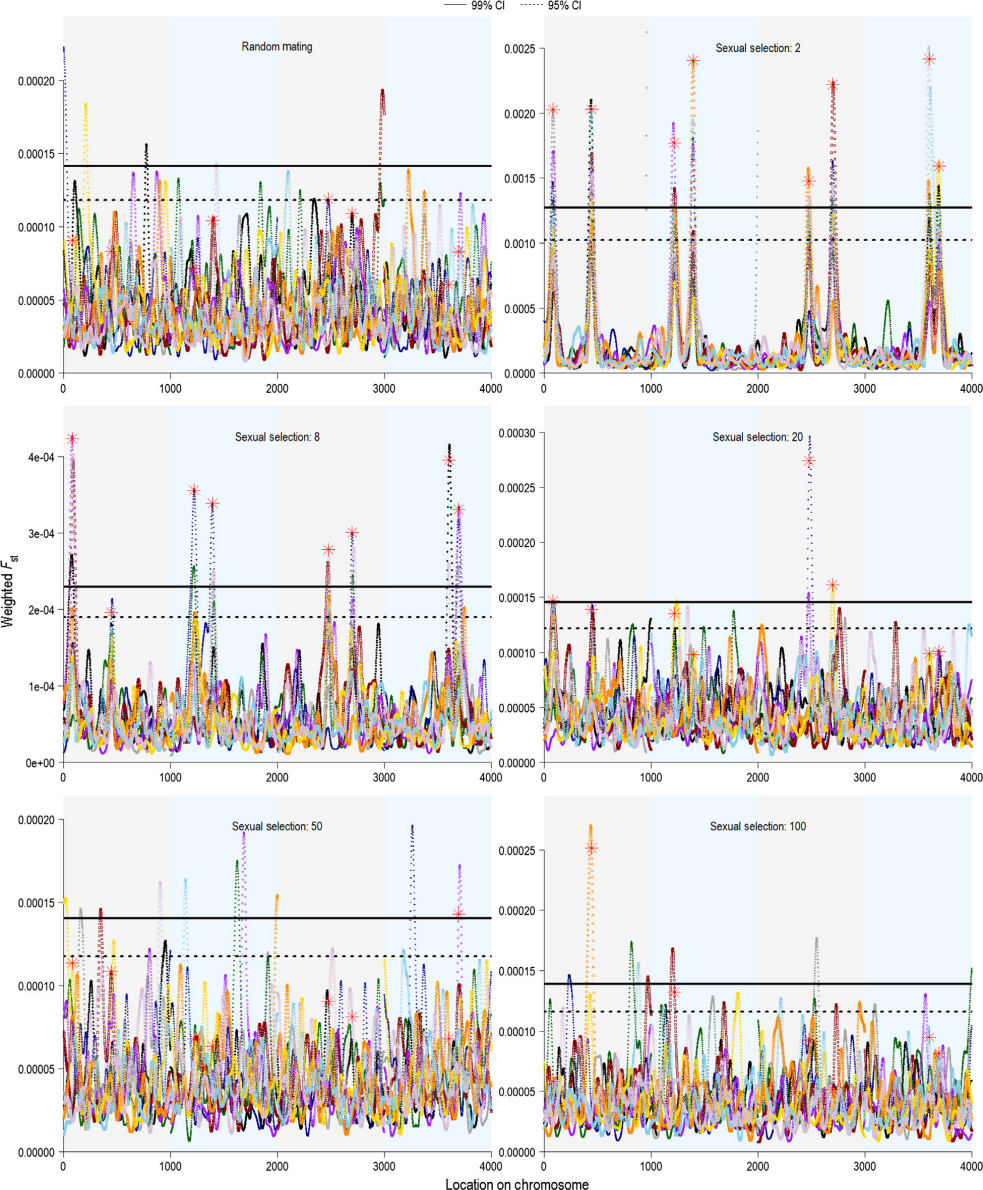
Sampling multiple populations improves the detection of real quantitative trait loci even when selection is weak. Each panel shows 10 replicates for that value of sexual selection strength. Each replicate is given a different color and shape. Both the 95% and 99% genome-wide confidence intervals are shown on the graphs, averaged across replicates.

We also explored whether multiple populations will help identify real quantitative trait loci even when sexual selection was weak. We found that with moderate sexual selection strengths 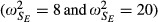, having multiple populations with the same quantitative trait loci helped validate peaks as real, and the locus of each spurious peak was restricted to a single population (Fig.13). In an empirical study, this approach would allow a researcher to focus on those outlier peaks that are present in multiple populations.

**Figure 14:**
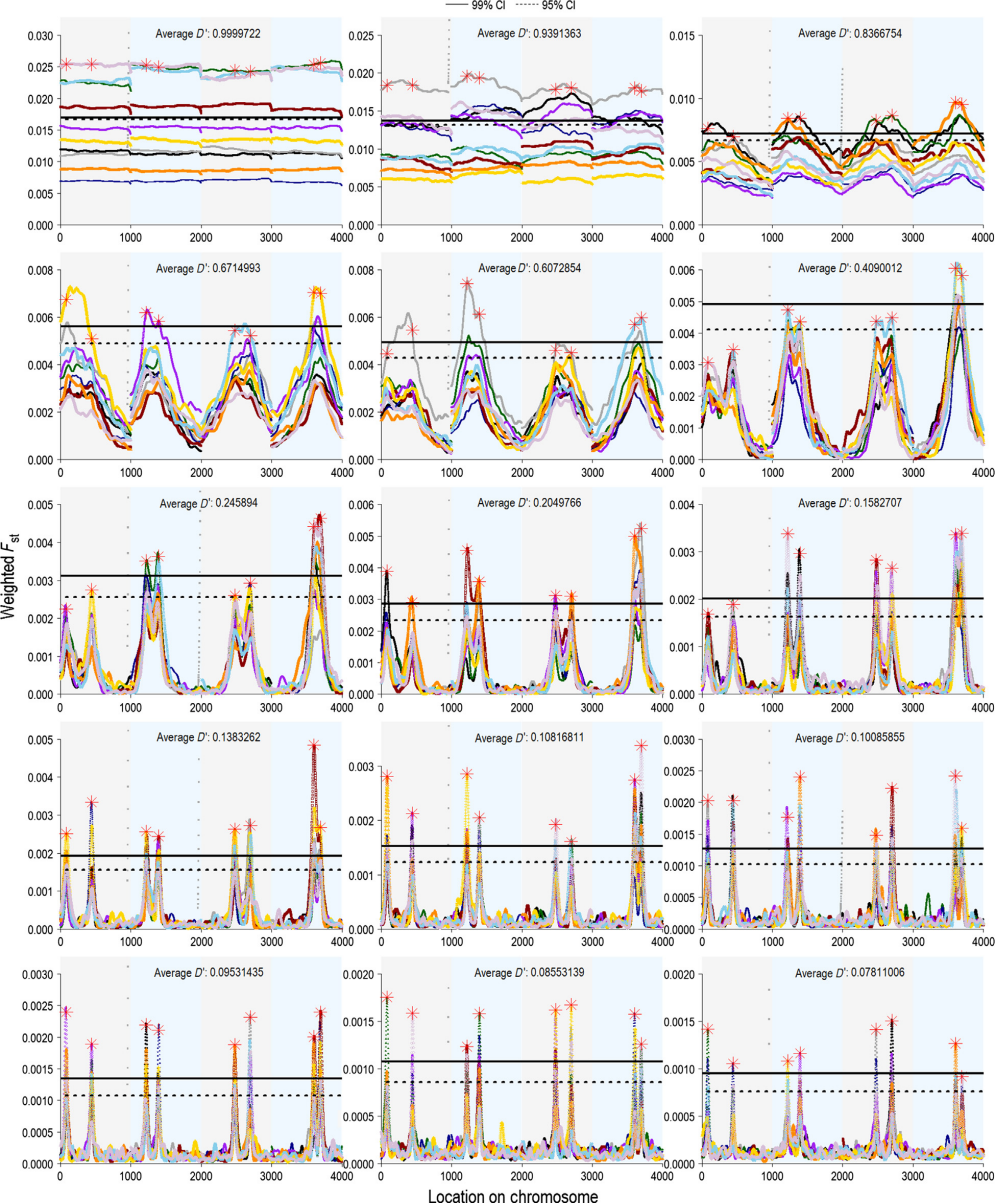
Detection of real quantitative trait loci is improved by sampling multiple populations at various levels of linkage disequilibrium, *D′*. Each panel shows 10 replicates for each value of *D′*, with a different color and shape for each replicate. Linkage disequilibrium was altered by changing the number of initial generations, and *D′* was calculated as the average per-chromosome pairwise linkage disequilibrium between 100 randomly chosen loci on a chromosome. We show the average 95% and 99% genome-wide confidence intervals.

When multiple populations were compared at different levels of linkage disequilibrium, the populations generally followed the same patterns. With only a few initial generations (1-2), there were no meaningful genome-wide patterns, as linkage disequilibrium was still nearly 1 (Fig.14). However, as linkage disequilibrium decayed with the addition of more initial generations, loci that were significant in one population were also often significant in others and contained a quantitative trait locus.

## Discussion

With this model, we set out to investigate the prospects for detecting a signature of sexual selection by comparing allele frequencies in adults and in offspring from one population using the type of data generated by next-generation sequencing approaches. We found that the genetic architecture of the trait was one of the most important factors determining the ability to detect selection. As expected, more real peaks were detected when fewer quantitative trait loci contributed to variation in the trait. When sexual selection was strong and acted on a phenotype determined by few quantitative trait loci, even small sample sizes (i.e., 100 parent–offspring pairs) could accurately detect some of the real quantitative trait loci without generating prohibitively huge numbers of spurious peaks. Overall, we showed that a genome-wide selection components analysis has the potential to detect signatures of sexual selection within a single population, at least in a best-case scenario with strong sexual selection, few loci of major effect, and optimal linkage disequilibrium.

The results from our simulations suggest that current empirical methods for assessing significance may be unreliable. The Benjamini–Hochberg false discovery rate, in particular, was unpredictable. Although the observed unreliability may be a feature of selection components analysis, this finding is worth noting, as many studies have used the false discovery rate in the analysis of RAD-seq and genome-wide scans (e.g., Hohenlohe et al. 2010a, 2013; Helyar et al. 2012; Narum et al. 2013). Another approach to determining outliers is to compare the overall distribution of *F*_ST_ values to a distribution from a set of putatively neutral loci from an empirical dataset to identify outlier loci (e.g., Lotterhos and Whitlock 2014). This approach requires a way to know which loci are likely to be neutral, however, and so is less versatile and may be more difficult to apply in RAD-seq studies of nonmodel organisms. We instead suggest that using the very simple measure of genome-wide confidence intervals, based on the empirical variance of the smoothed *F*_ST_ values, would not only be appropriate but also be an accurate and repeatable method for defining cutoff values, as it best excluded spuriously significant loci while catching the majority of real peaks in our model.

Even though the occurrence of spurious loci was rare in our model, spurious peaks occurred occasionally. In empirical studies, identifying which significant peaks are real and which are spurious may be challenging, but our results suggest that comparing multiple populations could help differentiate between real and spurious peaks. Even when genome scans are utilized to identify candidate regions that will later undergo further screening, it would be preferable to reduce the number of spurious loci detected to save time and resources. Although spurious loci occurred occasionally in our model, with multiple populations it was possible to identify the real peaks as those that occur consistently in all populations. Screening for peaks in multiple populations helped identify peaks when sexual selection was weak. This observation is consistent with recent evidence from empirical work that spurious loci should not be repeatable between populations or replicates. For instance, Tobler et al. (2014) showed that comparing replicates of laboratory-reared populations of *Drosophila melanogaster* was a very effective way to filter out false positives when looking for single nucleotide polymorphisms that responded to artificial selection regimes.

Environmental variation is expected to contribute to quantitative traits in real-world settings. We included environmental variation in our model and found encouraging results. Although large amounts of environmental variation dramatically reduced quantitative trait locus detection rates and increased the number of spurious loci, small amounts of environmental variation had very little effect on the detection of selection, even with many quantitative trait loci contributing to the trait. These small amounts of environmental variation (up to an environmental variance of 8) led to average heritability values within the range of 0.1 to 0.8, which is the range reported in studies with animal models (Visscher et al. 2008), so our genome-wide selection components analysis was robust to biologically relevant amounts of environmental variation.

Genome-wide selection components analysis was most effective at detecting only real peaks and not spurious ones when the sample size was large (>1000 adults and >1000 offspring). Such large samples may be difficult to collect and also would be very costly to genotype. Fortunately, even small sample sizes identified real quantitative trait loci, at least when sexual selection was strong, with little change in the average number of spurious loci detected: In a large population (carrying capacity = 10,000), sampling 100 adults and 100 offspring identified on average 14.5% of the quantitative trait loci (at least one real peak), but only detected 0.228 spurious peaks (less than one spurious peak, on average). If 100 parent–offspring pairs were sampled from each of two distant populations (assuming both populations had the same genetic architecture of the trait and similar selection pressures), then a real peak could be identified. This plan would not be logistically prohibitive, especially if other population genomics questions could be answered in the comparison of the two populations. Additionally, as many population genomics studies aim to identify candidate regions, it may be reasonable to use a less stringent cutoff than we used here (e.g., 90% instead of 99% confidence intervals), and increase the number of both real and spurious peaks detected.

Empirical work suggests that most quantitative traits in several model organisms appear to have many underlying quantitative trait loci of small effect (Flint and Mackay 2009). Our model’s ability to detect signatures of sexual selection was negatively impacted by an increase in the number of quantitative trait loci, which suggests that there may be limitations to the applicability of this method. However, we observed that a larger number of neutral markers included in our model increased detection rates of real peaks, suggesting that scanning a larger number of neutral markers may improve the ability to detect quantitative trait loci of smaller effect. Thus, genome-wide selection components analysis may indeed be able to capture signatures of selection on traits that are determined by many loci of small effect.

If researchers want to apply genome-wide selection components analysis to natural populations, we can provide several recommendations. First, this analysis was most effective in populations experiencing strong sexual selection, so it would be best applied to a species with clear evidence that sexual selection is occurring. Additionally, although larger sample sizes are always better, small sample sizes had the most spurious loci when the carrying capacity was small. Therefore, if the species of interest is known to have a small population size (as might be the case in some endangered species), investing in more comprehensive sampling may be especially worthwhile (although sampling may be invasive and could raise additional conservation concerns). Finally, it is important to note that we detected signatures of selection with this model and did not necessarily identify the exact locus underlying the trait. When we identified peaks as “real,” the quantitative trait locus had to be within 50 loci in either direction from the peak 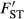 value. We tested different distances (peak detection widths, Fig.3) and found that any distance greater than 2 loci up to 100 loci had generally equivalent detection rates. In empirical work, the quantitative trait locus would be unknown, so additional work will probably be necessary in most cases to identify the actual DNA-level variant affecting fitness.

### Caveats

Several factors beyond the scope of this paper could affect genome-wide selection components analysis. First, our model of genetic architecture was not realistic in every way. For example, we did not incorporate variable recombination rates, although these are likely to occur in natural populations and could play a very important role in the effects of selection on the genetic architecture. It would be interesting to investigate how different patterns of recombination within the genome might affect selection components analysis. This could be done, for example, by making use of the *Drosophila* Genetic Reference Panel (Mackay et al. 2012; Huang et al. 2014), which encapsulates real patterns of recombination. Additionally, we allocated the same number of quantitative trait loci to each chromosome, which is an unlikely genetic architecture for a trait, and which may affect the ability of selection components analysis to identify real quantitative trait loci. Lastly, we implemented a contrived setup of our population to generate sufficient genetic variation before selection was imposed. We have also restricted our analysis to loci with relatively high minor allele frequencies in the population, even though quantitative trait loci could have small minor allele frequencies. A more realistic model might use coalescent simulations to generate uneven allele frequencies more commonly seen in the wild.

Additionally, viability selection was present in our model only to control the levels of additive genetic variation, so the model was not set up to address how strong viability selection might affect the power of selection components analysis, with or without strong sexual selection. However, investigating trade-offs between the strength of sexual and viability selection could be very interesting for further research. Also of interest would be the degree of the trade-off between natural and sexual selection. We set our natural selection optimum to 0 and sexual selection optimum to 4, so that trait values would be restrained. Testing different values of both optima could be very interesting and could result in intriguing changes at the genomic level. Similarly beyond the scope of this paper, but of great interest, is how genome-wide selection components analysis might be affected by viability selection on a trait separate from the target of sexual selection; that is, if sexual selection and viability selection both affected males, but acted on different traits with different genetic architectures.

In our model, the initial generations set up the population with genetic variation upon which sexual selection could act, so we did not model a long history of sexual selection in our simulated populations. Therefore, we could only address the case where sexual selection has been recently introduced to a system or where genetic variation is somehow maintained in the presence of strong sexual selection. We also did not investigate different types of mating systems, or those in which there are other costs to mating. We incorporated a cost of female choice (if females did not find an acceptable mate after sampling 50 males, they did not mate), but the impact of this parameter was not addressed in our analysis. There could be subtle effects of these details of the mating system on the detection of selection at the genomic level, but addressing how mating system parameters affect genome-wide selection components analysis is beyond the scope of this paper.

Although *F*_ST_ has been used as a measure of differentiation and population structure between populations, there is some murkiness surrounding its application between life stages within a population. More population genetics theoretical work is needed to evaluate whether there are any confounding factors in applying *F*_ST_ within a generation. Additionally, *F*_ST_ is sensitive to other modes of selection (e.g., background or positive selection), so it is possible that these forces may confound the effects of sexual selection. Although ideally we would be able to follow genotypes over multiple generations, *F*_ST_ is the most convenient summary statistic at this time. We believe that applying genome-wide selection components analysis using *F*_ST_ statistics to capture differentiation between life stages is the best method currently available, but that the same principle could be applied as genomics statistics are improved and refined.

In addition to these factors that we could not test with our model, our model is different from empirical studies in several ways. Importantly, empirical genomics studies would be affected by sources of error such as sampling bias and sequencing errors. We chose not to include these factors in our model, as these are problems that plague all current population genomics studies and have been evaluated by other researchers (e.g., Arnold et al. 2013; Davey et al. 2013; Gautier et al. 2013). Finally, what we present here can be called a best-case scenario, in which sexual selection is recent and strong, linkage disequilibrium and genetic variance are at ideal levels, and there are few quantitative trait loci with large effects.

## Conclusion

In summary, we investigated the potential for genome-wide selection components analysis to detect signatures of sexual selection using an individual-based simulation model, in which allele frequencies in adults and offspring from a single population were compared. We were able to accurately detect some or most of the quantitative trait loci underlying the trait under selection, even when sample sizes were low or the trait was highly polygenic. However, selection must be very strong and there must only be few loci of major effect for a high detection rate to occur. Implementation of this method in studies of natural populations could provide another tool to identify genomic regions that are affected by sexual selection, leading to a better understanding of how selection affects the phenotype and results in the heritable changes that allow evolutionary change in natural populations.

## Data Accessibility

Data and source code are archived on Dryad (doi: 10.5061/dryad.5k84d).
